# Effects of Temperament and Character Profiles on State and Trait Depression and Anxiety: A Prospective Study of a Japanese Youth Population

**DOI:** 10.1155/2012/604684

**Published:** 2012-08-22

**Authors:** Xi Lu, Zi Chen, Xiaoyi Cui, Masayo Uji, Wataru Miyazaki, Masako Oda, Toshiaki Nagata, Toshinori Kitamura, Takahiko Katoh

**Affiliations:** ^1^Department of Clinical Behavioural Sciences, Kumamoto University Graduate School of Life Sciences, 1-1-1 Honjo, Kumamoto 860-8555, Japan; ^2^Department of Public Health, Kumamoto University Graduate School of Life Sciences, 1-1-1 Honjou, Kumamoto 860-8555, Japan; ^3^Research Center of Applied Psychology, Chengdu Medical College, Chengdu, China; ^4^Kyushu University of Nursing and Social Welfare, Tamana 865-0062, Japan; ^5^Kitamura Institute of Mental Health Tokyo, Tokyo 107-0052, Japan; ^6^Department of Psychiatry, Graduate School of Medicine, Nagoya University, Nagoya 466-8550, Japan

## Abstract

*Objective*. To examine the effects of temperament and character profiles on state and trait depression and anxiety in a Japanese youth population. *Method*. Japanese university students were solicited for participation in a two-wave study, with assessments performed at Time 1 (T1) and Time 2 (T2), separated by a five-month interval. A total of 184 students completed the Japanese version of the temperament and character inventory (TCI) at T1 and the Hospital Anxiety and Depression Scale (HADS) at T1 and T2. We posited two latent variables, trait depression and anxiety, composed of the T1 and T2 HADS depression and anxiety scores, respectively. We also posited that temperament domain traits would predict character domain traits, and that all the personality traits would be linked to trait depression and anxiety and also predict T2 depression and anxiety. *Results*. Structural regression modeling showed that (1) only high Novelty Seeking predicted T2 Anxiety score, (2) trait depression and anxiety were linked to high harm avoidance and low self-directedness, and (3) trait depression was linked to high self-transcendence whereas trait anxiety was linked to low reward dependence, persistence, and cooperativeness. *Conclusion*. The characteristic associations between TCI subscales and depression and anxiety were limited to the trait rather than state aspects of depression and anxiety.

## 1. Introduction

### 1.1. Depression and Temperament and Character Domains

Since the introduction of the seven-factor model of personality [1] and the temperament and character inventory (TCI) [2], many investigations have examined the links between temperament and character traits of depression and anxiety. Most of these have demonstrated that individuals with depression score higher in harm avoidance (HA) and lower in self-directedness (SD) than those without depression. However, the majority of these reports used a cross-sectional research design [3–17]. Such studies are not free from state effects of depression on the self-report of the TCI. Several studies followed patients with depression before and after they achieved remission. Various investigations have reported increased postremission SD [18, 19], reduced HA [20], or both [21–24]. While in remission, however, patients with depression still showed higher HA [19, 20, 22, 25], lower SD [26, 27], or both [23, 24, 28–30] as compared with normal controls.

Changes in HA and SD scores before and after remission suggest that these TCI subscale scores can be influenced by the mood of the subject when filling in the questionnaire. However, significant differences in TCI scores between patients with depression and normal controls, even during remission, indicate that depression severity may consist of two components: one derived from the state-dependent effects of depression and the other from the effects of the intrinsic association between personality traits and depression.

This issue may be further clarified by longitudinal studies. Such studies can predict the onset of depression or depression severity at later stages of followup by using baseline TCI subscale scores after controlling for baseline depression severity. This may rule out the state dependency effects of TCI scores on current depression severity. For example, Naito, Kijima, and Kitamura [31] studied university students on two occasions with a three-month interval. They found that after controlling for T1 depression severity, depression at Time 2 could be explained by low SD scores at Time 1 but not by high HA scores. In a 12-month follow-up study, Cloninger, Svrakic, and Przybeck [32] found that high HA and low SD at Time 1 explained 44% of the variance in the change in depression between Time 1 and Time 2. Farmer and Seeley [33] conducted a four-year follow-up study of more than 500 community residents. They divided participants into those with depression and those without based on the cutoff point defined by the Center for Epidemiologic Studies Depression Scale. They found that participants with depression severity that was low at Time 1 but high at Time 2 scored lower in SD as well as reward dependence (RD), and cooperativeness (C) than those with low depression severity at both Times 1 and 2. Recently, Josefsson et al. [34] followed community residents for 10 years. They found that high HA scores at Time 1 predicted depression severity at Time 2. The literature thus suggests that depression severity may be associated with low SD and high HA.

### 1.2. Anxiety and the Temperament and Character Domains

Anxiety disorders have also been studied in terms of their relationship with TCI personality traits. People with panic disorder score high in HA and low in SD [35, 36]. Individuals with obsessive compulsive disorder are characterised by high HA [37–39], low SD [40], or both [12, 41–43], as well as, in a few studies, low NS [12, 41], low RD [43], or low C [12, 41–43]. Phobic disorders are also characterised by the combination of high HA and low SD [40, 44] in addition to low C and ST [45]. Specific phobias have been linked to high HA [40]. The combination of high HA and low SD is seen in people with posttraumatic stress disorder [46]. Anxiety symptoms in general have been linked to low SD [16] and high HA [10, 47].

### 1.3. Methodological Considerations

Although the abovementioned longitudinal studies elegantly controlled for the state dependency of TCI scores on depression severity by means of multiple regression analysis, they may not be free from flaws. Firstly, in such regression analyses, depression severity at Time 2 is primarily explained by that at Time 1, and only the remaining variance of the severity at Time 2 is explained by TCI scores. Hence, the portion of the covariance between depression and personality traits at Time 1 that is related to their intrinsic association may be treated as a part of state-dependent effects, resulting in possible underestimation of the real predictive power of personality traits on later depression severity. 

Second, these analyses are based on the assumption that depression severity is purely a “state” measure. There have been arguments that depression, like anxiety, may consist of trait and surplus components. The trait component is a temporally stable component reflecting enduring characteristics of individuals. The surplus component, on the other hand, is a variable one that reflects the current mood state. The state we observe is thus an amalgamation of these two components. Ritterband and Spielberger [48, 49] claimed that, like anxiety, depression is dividable into trait and surplus (which they termed “state”) components. Using the state depression and trait depression subscales of the Self-Analysis Questionnaire, Endler et al. [50] reported that the Beck Depression Inventory, a self-report measure of depression, was correlated more strongly with Trait-Depression subscale scores than state depression subscale scores. Nevertheless, the State-Trait Depression Scale [51] simply asks about participants' current (i.e., state) as well as usual (i.e., trait) mind condition. Hence the two may influence each other. We thought that it would be difficult for research participants to disentangle trait and surplus elements of their mood. Rather, we considered that the mood experienced by an individual at a given point in time could be statistically divided into the enduring trait component and the temporally changeable surplus component. This could be achieved by means of structural equation modeling (SEM), discussed later. 

Third, multiple regression analyses assume that all the variables are free of errors. This is an implausible assumption. Errors may blur what should otherwise be clearly observed. Thus, the addition of error variables in SEM may be a way to reduce such bias.

Fourth, past investigations have generally measured only depression. However, the oft-reported association between depression and anxiety suggests that caution is required when interpreting results concerning the association between depression and TCI subscale scores, because it may be confounded by anxiety scores.

Finally, many previous studies simultaneously entered TCI temperament and character subscale scores into the regression equation. However, a basic assumption of the psychobiology model of Cloninger, Svrakic, and Przybeck [1] is that temperament provides the basis for character development. Among Japanese populations [31, 52], for example, low HA is associated with high SD and C; high RD is associated with high C; high novelty seeking (NS) is associated with high self-transcendence (ST). Hence, the effects of a temperament trait such as high HA on depression may be either direct or mediated by a character trait such as reduced SD [53]. 

### 1.4. The Present Study

Taking into account these criticisms of longitudinal studies regarding the prediction of depression from baseline TCI scores, we conducted a study in which university students were twice administered questionnaires containing both depression and anxiety measures, separated by a five-month interval. 

**Figure 1:**
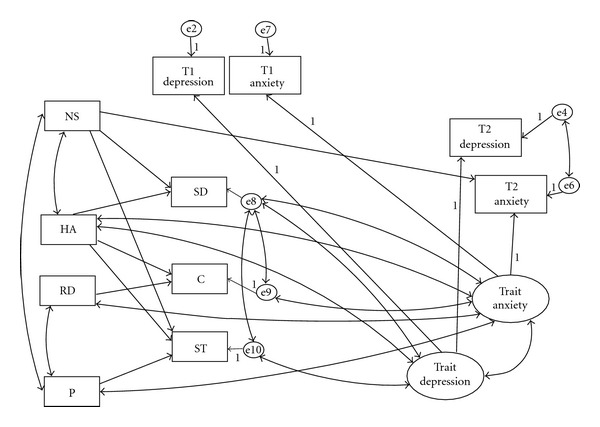
Structural regression path of the T1 HADS and T2 HADS dimensions and the TCI subscales. NS: novelty seeking; Ham: harm avoidance; RD: reward dependence; P: persistence; SD: self-directedness; C: co-operativeness; ST: self-transcendence.

In a structural equation model, we created latent variables comprised of trait components of both depression and anxiety (Figure 1). Observed (i.e., state) depression and anxiety scores consist of two components. One is the trait component, which is stable over the course of the investigation. The other is the surplus component, reflecting temporary ups and downs in mood. This component is unstable and is possibly responsive to the environment immediately before the assessment. We posited correlations between the trait depression and anxiety scores and the TCI subscale scores. This is because there are two alternative possibilities: mood effects on the self-report of the TCI and a real association between mood and personality. Then we posited that the three character subscale scores—SD, C, and ST—would be predicted by the four temperament subscale scores. This reflected the assumption that character develops based on temperament. Finally, we hypothesized that all seven TCI subscale scores would predict the states of depression and anxiety at Time 2.

## 2. Methods

### 2.1. Participants

University students in Kumamoto, Japan were solicited for participation in a two-wave study. Questionnaires were distributed to new students in May after they enrolled in college (T1) and again five months later (T2). At Time 1, 525 questionnaires were distributed out of which 240 (46%) usable questionnaires were returned. Of these students, 184 (77%) students responded at Time 2. The data of these 184 students were used for subsequent analyses. There were no differences between the students who responded at two occasions (*N* = 184) and who responded only at Time 1 (*N* = 56) in terms of age, gender, and the Hospital Anxiety and Depression Scale, and TCI subscale scores at Time 1 except for harm avoidance being slightly higher among those students who responded at the two measurement occasions. The 184 students' age ranged from 18 to 30 years old. These included 61 men and 123 women. The mean age (standard deviation) was 18.7 (1.0), with no difference observed between men and women.

### 2.2. Measurements

#### 2.2.1. Temperament and Character Dimensions

The TCI [1] was translated into Japanese [52] with the permission of Professor Cloninger. The Japanese items were retranslated back into English by an individual who was unaware of the original English in order for the original author to verify the wording. The TCI and its predecessor, the Tridimensional Personality Questionnaire, have been widely used both in Japanese patient and nonpatient populations [54, 55]. The TCI measures four temperament dimensions—NS, HA, RD, and Persistence (P)—and three character dimensions—SD, C, and ST. There are studies on the internal consistencies and factor structures of the Japanese versions [52, 56, 57]. These items were examined at T1.

#### 2.2.2. Depression and Anxiety

The Hospital Anxiety and Depression Scale (HADS [58]) is a self-report instrument designed to measure negative mood. The HADS consists of 14 items; the anxiety (HADS-A) and depression (HADS-D) subscales each include seven items. The psychometric properties of the Japanese version of the HADS [59] have been reported [60]. The HADS was included in the T1 and T2 surveys.

### 2.3. Statistical Analysis

We calculated the mean and standard deviation of each variable used in this study and examined the correlations between each pair of variables. We then constructed a path model using SEM based on our research hypotheses (Figure 1). We posited that (1) trait depression, a latent variable, would be composed of the T1 and T2 HADS-D; (2) trait anxiety, another latent variable, would be composed of the T1 and T2 HADS-A; (3) all the temperament subscales would be associated with all the character subscales; (4) all the TCI subscales would predict T2 depression and anxiety; (5) trait depression and anxiety would be associated with all the TCI subscales (or their error variables); (6) all the TCI subscales would be associated with T2 Depression and Anxiety; (7) trait depression and trait anxiety would be correlated with each other (Figure 1).

Covariances were added according to greater modification indices if such additions fit clinical and research assumptions. The fit of the model with the data was examined in terms of chi-squared (CMIN), comparative fit index (CFI), and root mean square error of approximation (RMSEA). According to conventional criteria, a good fit would be indicated by CMIN/df < 2, CFI > 0.97, and RMSEA < 0.05, and an acceptable fit by CMIN/df < 3, CFI > 0.95, and RMSEA < 0.08 [61]. All statistical analyses were conducted using the Statistical Package for Social Science (SPSS) version 19.0 and Amos 19.0. 

### 2.4. Ethical Considerations

This project was approved by the Ethical Committee of Kumamoto University Graduate School of Medical Sciences.

## 3. Results

The correlations between all variables used in the present study are shown in Table 1. In bivariate correlations, some of the TCI subscale scores were correlated with each other. Thus, high NS was associated with low HA, low P, low SD, and high ST. High HA was associated with low SD and low ST. High RD was associated with high P and high C. High P was associated with high ST. SD and C were correlated with each other.

T1 and T2 HADS-D and HADS-A were significantly correlated with HA and inversely with SD and C. In addition, T1 and T2 HADS-A were inversely correlated with RD. T1 HADS-A was correlated inversely with P, and T2 HADS-D was correlated with ST (Table 1).

Because there were significant correlations between many of the variables examined in this study, we created an original path model (Figure 1). According to the modification indices as well as theoretical considerations, we added correlations between NS and HA, between NA and P, and between RD and P. Correlations were also added between the error variables of SD and those of C and ST. 

**Figure 2:**
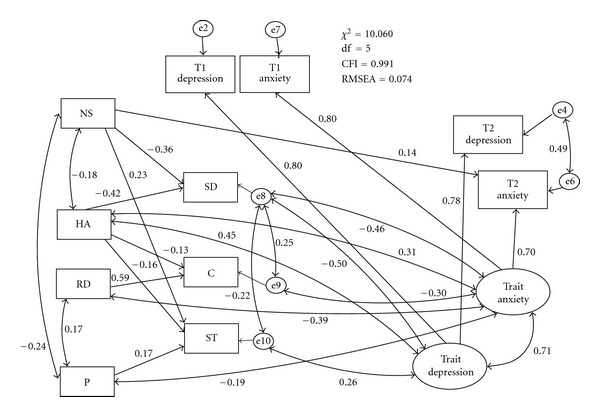
Final SEM model NS: novelty seeking; Ham: harm avoidance; RD: reward dependence; P: persistence; SD: self-directedness; C: cooperativeness; ST: self-transcendence. Paths without significance (<0.05) are not shown in the Figure (but were not deleted).

Our final path model can be regarded as good: CMIN/df = 2.01, CFI = 0.991, and RMSEA = 0.074 (Figure 2). As expected, scores of some temperament scales predicted those of character scales: significant paths were found from NS to SD and ST; from HA to SD, C and ST; from RD to C; from P towards ST. Several TCI scale scores were found to be associated with the scores of depression and anxiety traits: both trait depression and trait anxiety were associated with high HA and low SD. In addition, trait depression was associated with high ST whereas trait anxiety was associated with low RD, P, and C. Among the T2 HADS scores, only T2 anxiety scores were predicted by high NS.

## 4. Discussion

A primary finding of this longitudinal study was that it was not the surplus component of the mood measurement but rather trait depression and anxiety that were mainly associated with TCI subscale scores. Thus, trait depression was associated with high HA and low SD, as well as high ST. Trait anxiety was similarly associated with not only high HA and low SD, but also with low RD, P, and C.

People with high HA and low SD may be characterised by depressive and anxious traits that are part of their personalities. Such people may be more likely to develop clinical mood and anxiety disorders under stressful life situations. Previous studies have found links between high HA and low SD on the one hand and mood and depressive disorders on the other, but these findings may be biased by the fact that trait and surplus components were measured simultaneously as state components.

In this study, we observed a significant link between trait depression and high ST. This link has not been consistently reported. However, some investigators have found that people with bipolar disorder were characterised by high ST in addition to high HA and low SD [13, 26, 62, 63]. ST is a character dimension that is associated with spirituality. This is an adaptive personality trait if combined with high SD and C, but is otherwise suggestive of schizotypal personality [1]. The slight but significant association between trait depression and ST in this study suggests that some students high in ST are prone to the future development of bipolar disorder. 

Unlike trait depression, trait anxiety was also associated with low RD and C. It has been reported that low RD and C are characteristics of anxiety disorders such as obsessive compulsive and phobic disorders. The results of the present study are in line with these findings. People who are low in RD are practical and cold and can be withdrawn and detached. People who are low in C are socially intolerant, critical, revengeful, and destructive and may thus be more anxious when relating to others. We consider high HA and low SD to be personality traits that are related to dysphoric mood in general, including depression and anxiety, whereas low RD and C are specific to anxiety.

Strengths of the present study include a longitudinal research design and the statistical separation of the trait and surplus components of depression and anxiety. Past studies have usually treated scores of mood measures as state indicators. In our view, they fail to distinguish the temporary reactive components of dysphoric mood from the stable ones. The former are more likely to be induced and maintained by immediate environmental factors such as stressful life events and enacted social support. In contrast, the latter is more likely to be dispositional. Our finding that TCI profiles were associated with trait components is in agreement with this notion. We expect that reanalysing the data produced by past investigations using our current statistical methods may cast more light on this issue.

Limitations of the present study should be noted. First, our sample size was modest. Given that SEM is a statistical method that requires a large sample size, further studies using a larger sample size are necessary. However, the good fit of the model to the relatively small current sample is encouraging. Second, we used a university student population. University students between 18 to 30 years of age constitute the study population. A few students were out of range of youth in this study. Nevertheless, the age range was narrow, and we should not extrapolate the data to older populations, particularly since TCI subscale scores vary with age [2]. Our approach distinguishing trait and surplus components should also be used to study clinical populations. The influence of mood state on the self-report of personality may be greater in a clinical population than a nonclinical population. We did not use diagnostic categories such as major depressive episode or generalized anxiety disorder. The use of structured diagnostic interviews will be necessary in further studies. A fourth shortcoming of the present study was that we assessed subjects at only two time points, and the time interval between the two assessments was five months. More assessment points may be required if we are to employ sophisticated statistical methods, for example, growth curve models. Furthermore, different results may emerge if a longer followup is employed.

The links between TCI subscales and trait depression and anxiety are the result of an inherent association combined with the effects of mood state on the self-report of personality. This issue is difficult to disentangle using the research design employed in the present study. One possible way to cast light on the distinction between the two components is to use multiple raters of each individual's personality, for instance the participant him- or herself as well as those who know him or her well (e.g., family members and friends). This approach is beyond the scope of the present study but would benefit from further study.

Taking into consideration these shortcomings, the present research showed that TCI profiles were associated with the trait components of depression and anxiety rather than their surplus components. Depression and anxiety traits shared several of the same TCI characteristics, including high HA and low SD, but differed in specific details, for instance with low C and RD being associated with anxiety only.

## Figures and Tables

**Table 1:** Correlations between variables used in the path analysis (*N* = 184).

	1	2	3	4	5	6	7	8	9	10	11	12	13
(1) Gender (male—1; female—2)	—												
(2) Age	−0.09	—											
(3) NS	−0.08	0.03	—										
(4) HA	0.06	−0.02	−0.19*	—									
(5) RD	0.38**	0.07	0.14	0.08	—								
(6) P	0.09	0.00	−0.19*	−0.05	0.15*	—							
(7) SD	−0.02	−0.06	−0.25**	−0.36**	0.02	0.09	—						
(8) C	0.36**	0.07	0.01	0.07	0.57**	0.13	0.24**	—					
(9) ST	0.09	−0.07	0.20**	−0.23**	0.09	0.15*	−0.14	0.13	—				
(10) T1 HADS-D	0.07	0.03	0.10	0.35**	−0.08	−0.05	−0.54**	−0.18*	0.13	—			
(11) T1 HADS-A	−0.07	0.01	−0.01	0.24**	−0.30**	−0.15*	−0.43**	−0.40**	0.01	0.53**	—		
(12) T2 HADS-D	0.02	0.05	0.11	0.24**	−0.11	0.06	−0.43**	−0.16*	0.15*	0.56**	0.34**	—	
(13) T2 HADS-A	−0.10	−0.01	0.10	0.28**	−0.28**	−0.11	−0.43**	−0.32**	0.03	0.48**	0.56**	0.61**	—
Mean	1.67	18.3	26.3	34.9	31.6	16.6	39.0	50.5	17.1	5.4	4.3	5.5	4.6
Standard deviation	0.47	0.9	6.5	7.4	6.0	3.7	8.1	7.8	6.4	3.6	2.8	3.7	3.2
Cronbach alpha	—	—	0.72	0.78	0.72	0.62	0.78	0.82	0.80	0.78	0.61	0.76	0.69

NS: novelty seeking; HA: harm avoidance; RD: reward dependence; P: persistence; SD: self-directedness; C: cooperativeness; ST: self-transcendence. *P < 0.05; **P < 0.0.
